# The Clifford Allbutt Presentation

**Published:** 1919-06-21

**Authors:** 


### THE CLIFFORD ALLBUTT PRESENTATION.

A few weeks ago a project was set on foot for a presentation portrait of Sir T. Clifford Allbutt, F.R.C.P., F.R.S., Regius Professor of Physic at Cambridge University and Chairman of the British Medical Association. Backed by the latter, to which for many years, and especially during the War, Sir Clifford has rendered sterling services, as well as by other representative corporate bodies of the medical profession, there can be no doubt as to the response that is being and will be made to this appeal. Apart from that, amongst living British physicians still on the active list Sir Clifford holds an unique position for professional reputation, admitted scholarship, multifarity of activities, and world-wide prestige, both in the profession and outside it. Few, if any, can be said to equal or even to approach the record of this distinguished man of science. At least nine universities within the Empire have conferred honorary degrees upon him; and the debt that his own *alma mater* owes him for the development of the Cambridge Medical School this last twenty-seven years is one that no words can adequately describe or acknowledge. As Regius Professor of Physic the direction of the Medical School was for years practically in his hands during the long interregnum that followed the death of Sir George Humphry, Regius Professor of Surgery, in the early 'nineties; nor is it any disparagement of the holders of this latter post in recent years to suggest that their influence upon the profession at large and the Cambridge medical graduates in particular has not equalled that of the wonderful veteran whom the Royal Colleges no less than the British Medical Association are now delighting to honour.

To pile superlatives is only too easy when dealing with a leader so admired and respected as the subject of this article. Let us therefore simply record some of the facts of his career. He graduated at Cambridge some sixty years ago, and will shortly be eighty-three years of age. He invented the "short" clinical thermometer, which generations of medical men have now been using without suspecting that the originator of this familiar tool might still be alive and in harness. He is Consulting Physician to the Leeds Infirmary as well as to two or three London hospitals. He was a Commissioner in Lunacy from 1889 to 1892, when he became Professor at Cambridge, and of course also Physician to Addenbrooke's Hospital. For width and depth of culture, and for real scholarship, no less brilliant than painstaking, Sir Clifford Allbutt has few, if any, equals that we know of in the medical profession. And this amazing octogenarian has actually added to his commitments during the Great

—perhaps by the time this appears in print it may be also the late—War. On the Central Medical War Committee, as at the head of the British Medical Association, Sir Clifford has been by no means a figurehead, but has never spared himself in tasks which bring little recognition and no gratitude.

To speak of his contributions to the literature of medicine is almost superfluous; but to show the Professor's versatility it may be mentioned that nearly fifty years ago he published a book on the use of the ophthalmoscope, while for years he has been, perhaps, the keenest student and most elegant expounder of the special arterial changes which accompany advancing years, changes which have certainly been kept most successfully at bay in his own arterial system. Nor should mention be omitted of Sir Clifford's contributions on medical subjects to the columns of The Hospital, upon which were bestowed the same care and incessant striving after the perfect phrase and the lucid exposition that always characterise his writings. To this Nestor of the profession, this ripe and sage counsellor, the honour of a presentation portrait can be but one, and that a small, incident in a career fuller of tokens of esteem than any the present generation of medical men can remember. As a climax to a life, so full and varied, it has a special appropriateness which will perhaps recommend it to Sir Clifford Allbutt. It will be a tribute from all sections of his profession, and from the hosts of individual friends and pupils by whom it is being subscribed.

